# Subtoxic Concentrations of Hepatotoxic Drugs Lead to Kupffer Cell Activation in a Human *In Vitro* Liver Model: An Approach to Study DILI

**DOI:** 10.1155/2015/640631

**Published:** 2015-09-28

**Authors:** Victoria Kegel, Elisa Pfeiffer, Britta Burkhardt, Jia L. Liu, Katrin Zeilinger, Andreas K. Nüssler, Daniel Seehofer, Georg Damm

**Affiliations:** ^1^Department of General, Visceral and Transplantation Surgery, Charité-University Medicine Berlin, Augustenburger Platz 1, 13353 Berlin, Germany; ^2^BG Trauma Center, Siegfried Weller Institute, Eberhard Karls University Tübingen, Schnarrenbergstrasse 95, 72076 Tübingen, Germany; ^3^Bioreactor Group, Berlin-Brandenburg Centre for Regenerative Therapies (BCRT), Charité-University Medicine Berlin, Augustenburger Platz 1, 13353 Berlin, Germany

## Abstract

Drug induced liver injury (DILI) is an idiosyncratic adverse drug reaction leading to severe liver damage. Kupffer cells (KC) sense hepatic tissue stress/damage and therefore could be a tool for the estimation of consequent effects associated with DILI. Aim of the present study was to establish a human *in vitro* liver model for the investigation of immune-mediated signaling in the pathogenesis of DILI. Hepatocytes and KC were isolated from human liver specimens. The isolated KC yield was 1.2 ± 0.9 × 10^6^ cells/g liver tissue with a purity of >80%. KC activation was investigated by the measurement of reactive oxygen intermediates (ROI, DCF assay) and cell activity (XTT assay). The initial KC activation levels showed broad donor variability. Additional activation of KC using supernatants of hepatocytes treated with hepatotoxic drugs increased KC activity and led to donor-dependent changes in the formation of ROI compared to KC incubated with supernatants from untreated hepatocytes. Additionally, a compound- and donor-dependent increase in proinflammatory cytokines or in anti-inflammatory cytokines was detected. In conclusion, KC related immune signaling in hepatotoxicity was successfully determined in a newly established *in vitro* liver model. KC were able to detect hepatocyte stress/damage and to transmit a donor- and compound-dependent immune response via cytokine production.

## 1. Introduction

Drug induced liver injury (DILI) represents an idiosyncratic adverse drug reaction responsible for severe patient morbidity and mortality and in consequence for the withdrawal of about 20% of new drugs from the market [1, 2]. In the USA, about 60% of all cases of acute liver failure and 10–20% of fulminant or subfulminant hepatitis originate from drug toxicity [3, 4]. Milder forms of DILI are assumed to occur in a high number of unknown cases. Therefore, the incidence and prevalence of DILI are only partially known [5]. The idiosyncratic origin of DILI and its unspecific reactions are the reason why it is still difficult to predict the potential risk of DILI in preclinical drug testing [6].* In vivo* animal studies are not suitable for reflecting the idiosyncratic nature of DILI and its low frequency would require very high numbers of animals to detect DILI events [2, 7].

Additionally, the occurrence of immune tolerance reactions in the liver can influence DILI consequences* in vivo* [8].* In vitro* studies using human cells could bypass systemic tolerance reactions and thus better reflect the human situation. However, it is known that many* in vivo* hepatotoxic effects are not detected in primary human hepatocytes (PHH) monocultures, which are however considered to be the gold standard of* in vitro* liver models. The lack of a physiological 3D environment and the absence of nonparenchymal cells are discussed as possible reasons for an insufficient reflection of DILI mechanisms in conventional 2D hepatocyte cultures [9–11].

The mechanisms of DILI are not yet sufficiently clarified. According to different hypotheses, an immune-mediated mechanism is considered to be a major factor in its pathogenesis [2, 12, 13]. This mechanism of action starts with the hepatic biotransformation of drugs, which can lead to the production of reactive metabolites [14]. Hydroxylation by cytochrome P450 enzymes especially can produce hydroquinone, benzoquinoneimine, and catechol structures, which are of electrophilic nature. Such compounds disturb the redox balance and induce the generation of reactive oxygen species (ROS) leading to oxidative stress. Additionally, electrophilic metabolites can react with endogenous nucleophilic groups of DNA and proteins. The reaction with proteins leads to the formation of haptens. If released, these haptens can be identified by immune cells due to their antigenic character [12, 13], causing sensitizing reactions or, at worst, the induction of autoimmune diseases [15].

Kupffer cells (KC) are the primary macrophage population of the liver. They are on the one hand part of the scavenger system, which is responsible for systemic blood clearance and on the other hand responsible for detection of local tissue damage. In this function, KC are active in phagocytosis of cell debris, soluble macromolecules, and colloids as well as endogenous and foreign proteins [16]. Besides the recognition of cellular stress and cell death in hepatotoxic events, KC also fulfill a transmitter role in the communication to the immune system by antigen presentation and cytokine secretion [17]. KC activation by lipopolysaccharides (LPS), cell debris, haptens, or cytokines is accompanied by intracellular activation of the NF-*κ*B signaling pathway mediated by reactive oxygen intermediates (ROI) [18]. Once activated, KC can differentiate into M1 type and M2 type macrophages depending on the signals received and on the genetic background. M1-KC play an important role in innate immunity and proinflammatory reactions. This inflammatory cell type is supported by TH1 cells. The alternative M2 response is depending on TH2 cells and ends in tissue-protective reactions. M2-KC promote maturation and activation of other KC, enhance tissue repair, and have a beneficial effect on vascular growth and nutrient homeostasis [19, 20]. Each KC class is associated with specific cytokines. M1-KC produce the proinflammatory cytokines IL-6, IL-8, and TNF-*α*, while M2-KC are associated with the anti-inflammatory cytokines IL-4, IL-5, and IL-10 [21–23]. Additionally, prostaglandin E_2_ (PGE-2) can be released, which inhibits TNF-*α* and IL-6 production by KC in an autocrine feedback loop and attenuates the induction of acute-phase proteins [24]. PGE-2 is therefore associated with the M2-KC response rather than with the M1-KC response.

Two well-known hepatotoxic compounds responsible for the induction of DILI are acetaminophen (APAP) and diclofenac (DIC) [3]. Both compounds are nonsteroidal anti-inflammatory drugs (NSAID). APAP is transformed by cytochrome P450 (CYP) 2E1 and CYP1A1 to the reactive metabolite N-acetyl-p-benzoquinone imine [23]. APAP is known for the induction of hepatic oxidative stress and in consequence glutathione depletion leading to acute liver failure. Protein adduct formation has also been described but plays a minor role in APAP hepatotoxicity [25]. DIC is metabolized by CYP2C9 and CYP3A4 to two hydroxylated metabolites consequently transformed in secondary reactions into metabolites with benzoquinone imine structure [26]. Both metabolites have been shown to react with proteins. Hapten formation correlates with the occurrence of sensitization reactions towards DIC. Beside hapten formation, the generation of ROS is also described but is of minor clinic relevance compared to APAP [23].

Aim of the present study was the establishment of a human* in vitro* KC culture model for the investigation of immune-mediated signaling in hepatic pro- and anti-inflammatory reactions involved in the pathogenesis of DILI.

For the present study, PHH and KC were isolated from human liver resectates using a two-step collagenase perfusion technique followed by selective separation steps to get purified PHH and KC fractions. KC were identified and characterized by morphological und functional investigations. Optimization of KC culture conditions allowed for a cultivation for up to 5 d. The known hepatotoxic drugs APAP and DIC were used at subtoxic concentrations to simulate a DILI-like event in PHH cultures. Supernatants of drug-treated PHH were then used to stimulate KC cultures. While most liver models for hepatotoxicity testing usually need high concentrations leading to definite toxic effects, this new model allows for detecting hepatotoxic cell stress also in a subtoxic concentration range.

## 2. Material and Methods

### 2.1. Chemicals

The hepatocyte culture medium was based on Williams' Medium E with GlutaMAX (Gibco, Paisley, UK), supplemented with 10% FCS (Gibco), 32 mU/mL Insulin (Sanofi Aventis, Frankfurt am Main, Germany), 15 mM HEPES, 0.1 mM MEM NEAA (100×), 1 mM pyruvate (all by Gibco), and 1 mg/L dexamethasone (Fortecortin, Merck, Darmstadt, Germany).

KC culture medium was based on RPMI low glucose (GE Healthcare, Pasching, Austria) supplemented with 10% FCS, 1% L-glutamine, and 6.3 mM N-acetyl-L-cysteine (all by Gibco). KC starvation medium was based on RPMI low glucose supplemented with 1% L-glutamine. All media were supplemented with 100 U/100 *μ*M penicillin/streptomycin (Gibco) prior to use.

PBS was purchased from Gibco. Percoll, Trypan Blue, and Hanks Balanced Salt Solution (HBSS) were provided by Biochrom (Berlin, Germany). All other chemicals were purchased from Sigma (Munich, Germany), if not stated differently.

### 2.2. Isolation and Culture of Primary Human Hepatocytes and Kupffer Cells

For mimicking immune-mediated reactions in DILI, a human* in vitro* liver model based on primary human liver cells was established. PHH and KC were isolated in parallel from the same donor tissue to avoid immune reactions due to incompatibility.

PHH and KC were isolated from nontumorous human liver tissue, which remained after partial liver resection in patients with primary or secondary liver tumors. Additionally, corresponding human blood samples were obtained and used for production of autologous serum. Informed consent of the patients was obtained according to the ethical guidelines of the Charité-Universitätsmedizin Berlin.

PHH were isolated by a two-step collagenase perfusion technique according to Nüssler et al. [27]. PHH contained in the gained cell suspension were enriched by double centrifugation at 50 ×g, 5 min, 4°C. The pellet was suspended in hepatocyte culture medium and seeded at a density of 145,000/cm^2^ in cell culture plates. Culture medium exchange was performed 12 h after seeding and afterwards every 24 h. Prior to starting APAP or DIC treatment, the culture medium was exchanged against hepatocyte starvation medium.

The supernatant of the initial centrifugation of the cell suspension was used for KC isolation [28]. To eliminate remaining erythrocytes, the supernatant was centrifuged at 72 ×g, 5 min, 4°C. The supernatant, which contained the nonparenchymal liver cells (NPC), was centrifuged at 650 ×g, 7 min, 4°C. The pellet consisting of KC, hepatic stellate cells, and liver endothelial cells was resuspended in 20 mL HBSS. For enrichment of KC, the cell suspension was subjected to a Percoll density gradient centrifugation. A two-level gradient consisting of a 25% Percoll solution on top of a 50% Percoll solution was prepared. The cell suspension was carefully placed on top of the 25% Percoll gradient and centrifuged at 1800 ×g, 15 min, 4°C, without brake. The cells in the interphase between 25% and 50% Percoll were collected, washed once with HBSS, and resuspended in KC starvation medium. The cell number and viability of the contained KC were determined by using the Trypan blue exclusion technique. To remove remaining NPC, the selective adherence capacity of KC on cell culture plastics was used. KC were seeded at a density of 0.5 × 10^6^ cells/cm^2^ on a 24-tissue culture plate (Falcon BD, Heidelberg, Germany) and cultured for 25 min at 37°C, 5% CO_2_ in a humidified incubator. Not adhered NPC were removed by washing the culture plate with HBSS. KC were then maintained in KC culture medium for at least 12 h overnight. The medium was replaced by KC starvation medium on the next day and KC were cultured at least for 4 h in that medium before the cells were used for experiments.

### 2.3. Optimization of Culture Conditions

Previous studies had shown that KC isolated from human liver tissue show a donor-dependent initial activation [29]. Due to potentially varying initial KC activation levels, we performed experiments designed to reduce or stabilize the KC activation. KC are activated by different factors like endogenous or foreign proteins, LPS, or environmental changes.

To exclude additional KC activation by xenogenous proteins contained in fetal calf serum, cultivation in KC culture medium with autologous serum or without any serum was tested. Autologous serum was generated by centrifuging 10 mL blood from the patient at 1000 ×g, 10 min and 4°C.

Additionally, a potential reduction of the initial KC activation by addition of antioxidants to KC cultures was investigated. Therefore, KC were cultured for up to 108 h by using KC culture medium supplemented with or without 10 mM *n*-acetylcysteine or 10 mM ascorbic acid.

### 2.4. Characterization of KC

#### 2.4.1. Immunofluorescence Staining

The purity of the isolated KC was determined by immunofluorescence staining of CD68, which is a surface protein of the macrophage lineage, including monocytes, histiocytes, giant cells, KC, and osteoclasts. An antibody against CD68 (R&D Systems, Minneapolis, USA) and a secondary antibody coupled with phycoerythrin (PE) (Santa Cruz Biotechnology Inc., Heidelberg, Germany) were used for staining.

Additionally, the ability for phagocytosis was evaluated by using FITS coupled latex beads (FluorisBite plain YG3.0 microspheres, Polyscience). Cell nuclei were stained with Hoechst 33342 (Sigma-Aldrich GmbH, St. Louis, US). Images were taken with a fluorescence microscope (Zeiss, Jena, Germany).

#### 2.4.2. Cell Viability/Cell Activity

In order to evaluate the cell viability as well as changes in energy metabolism, the cell activity was determined using the XTT assay (Roche Diagnostics GmbH, Mannheim, Germany). The test was performed according to the manufacturer's protocol. After 2 h incubation time, the supernatants were transferred into a 96-well plate and the absorbance was measured at 492 nm in a microplate reader (FLUOstar OPTIMA, BMG Labtech, Ortenberg, Germany).

#### 2.4.3. Measurement of Intracellular Reactive Oxygen Intermediates (ROI)

ROI play an essential role in signaling pathways of inflammatory reactions. Therefore, the formation of intracellular ROI was measured by using the fluorogenic substance dichlorodihydrofluorescein diacetate (DCF-DA) according to [30] with minor modifications. The cell-permeable DCF-DA diffuses into cells and is deacetylated by cellular esterases and oxidized by ROI to dichlorodihydrofluorescein (DCF). For ROS measurement, the culture medium was replaced with RPMI medium without serum and phenol red, but containing 20 *μ*M DCF-DA (Santa Cruz Biotechnology, Inc., Heidelberg, Germany) followed by incubation for 30 min at 37°C, 5% CO_2_ in a humidified incubator. Subsequently, the supernatants were aspirated and the cells were incubated with fresh medium without serum and phenol red for 1 h. Fluorescence was measured in a microplate reader at an excitation wavelength of 492 nm and an emission detection of 520 nm.

### 2.5. Kupffer Cell Stimulation

Selective activation of KC was performed by incubation with hepatotoxic drugs (APAP, DIC) or using lipopolysaccharide (LPS) stimulation. For equilibration of the KC, cells were cultured in starvation medium for 24 h. Subsequently, KC were incubated with 100 *μ*M APAP, 100 *μ*M DIC, or with different LPS concentrations in starvation medium for further 24 h. KC activation was determined by the measurement of ROI formation and cell viability as described above.

To investigate the activation of KC following hepatocyte damage, KC were incubated with the supernatants of drug-treated PHH from the same donor. Therefore, the PHH were cultured for 4 h and KC for 5 h in starvation medium for equilibration of the cells. Subsequently, the PHH were stimulated with 100 *μ*M APAP or 100 *μ*M DIC in starvation medium for 1 h, respectively. Then, the supernatants of the compound-treated PHH were transferred onto the KC. After 2 h incubation time, ROI formation and cell activity were measured as described above. Additionally, the supernatants were collected and stored at −80°C after freezing in liquid nitrogen for subsequent measurement of pro- and anti-inflammatory cytokine formation as marker for KC response. These experiments were performed with cells from three independent donors and evaluated individually for each donor (Table 1).

### 2.6. Cytokine ELISA

In order to evaluate inflammatory reactions of KC, the formation of pro- and anti-inflammatory cytokines was investigated. Tumor necrosis factor-*α* (TNF-*α*), interleukin-6 (IL-6), and interleukin-10 (IL-10) ELISA Kits (PeproTech GmbH, Hamburg, Germany) as well as a prostaglandin E2 (PGE-2) ELISA Kit (Thermo Fischer Scientific, Waltham, USA) were used for the measurement of cytokine concentrations in cell culture supernatants. Cytokine formation was measured following the manufacturer's instruction.

### 2.7. Statistical Analysis

Data were analyzed by one-way or two-way ANOVA, with a *t*-test or a Mann-Whitney test using Graph Pad Prism 5 software. Results are given as means ± SEM or as median including the interquartile range, minimum and maximum values presented as box plots. Differences were considered as significant at *P* < 0.05. Only data from experiments performed at least three times with cells from different donors were subjected to statistical analysis.

## 3. Results

### 3.1. Isolation and Characterization of PHH and KC

**Figure 1:**
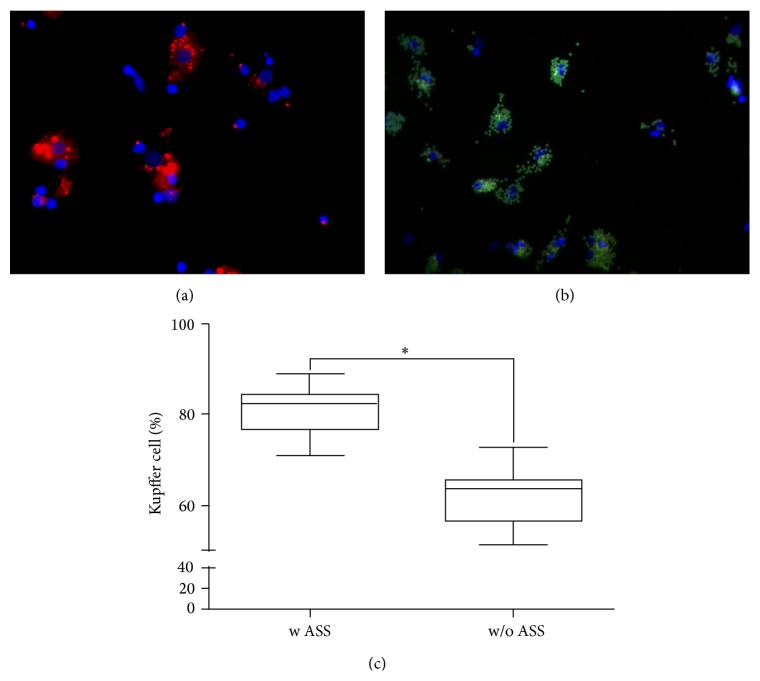
*Identification of KC and determination of the purity of KC cultures*. KC were identified by IF staining for CD68 (a) and the phagocytosis of fluorescent latex beads (b). The percentages of CD68-positive and phagocytosis-positive cells isolated with an adherence separation step (w ASS) or without an adherence separation step (w/o ASS) are shown in (c). Data are shown as box plots, representing the median, the interquartile range, and minimum and maximum values. ∗ At least *P* ≤ 0.0001 (unpaired *t*-test), *N* = 3, *n* = 9.

KC were successfully isolated from 37 different donors using the supernatants remaining from PHH isolation. Trypan blue staining showed that >90% of KC were viable. A yield of 1.2 ± 0.9 × 10^6^ KC per gram of liver tissue was obtained. KC were identified by immunostaining of the macrophage-specific surface protein CD68 and by their ability for phagocytosis of fluorescent latex beads (Figure 1(a)). CD68-positive and phagocytosis-positive cells were counted in relation to total cells stained with Hoechst dye (Figure 1(b)). A purity of 60% was obtained in the KC-rich cell suspension. The performance of the adherence separation step increased the purity to >80% (Figure 1(c)).

**Figure 2:**
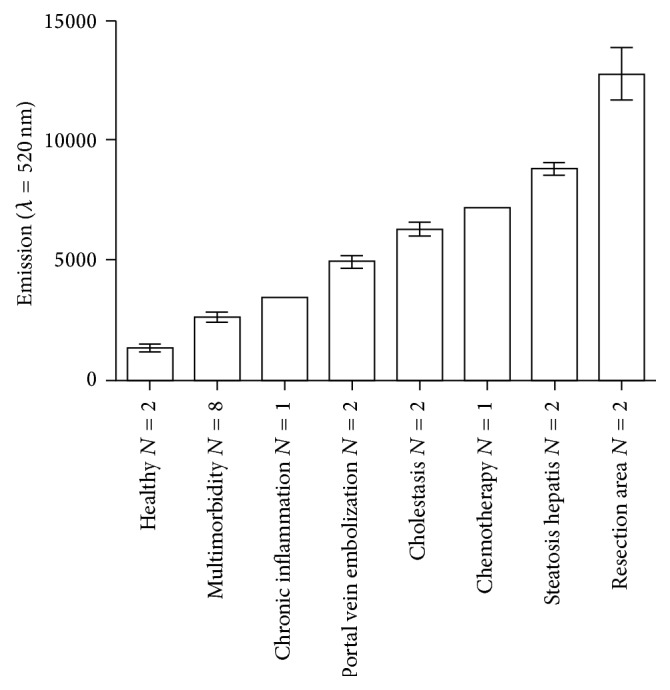
*Initial KC activation*. Initial ROI levels correlated with tissue quality and donor anamnesis. Initial ROI levels were measured in KC cultures by means of the DCF assay performed directly after KC isolation. Data show means ± SEM. *N*: shown in the figure.

The determination of KC activity was performed directly after the isolation procedure. Intracellular ROI levels as mediator in the NF-*κ*B signaling pathway were measured by the DCF assay. The measurement of the initial KC activation revealed variable intracellular ROI concentrations in cultures from donors with different donor anamnesis and tissue quality (Figure 2, Table 1). The lowest KC activity was detected in healthy patients with low BMI and early tumor stages (considered as healthy tissue). Patients with multimorbidity or chronic inflammation showed preexisting moderate KC activation as an indicator of chronic cell stress/damage. In contrast, the KC from livers with tissue damage caused by portal vein embolization, cholestasis, or recently performed chemotherapy revealed the highest KC activation levels. The same was true for steatotic liver or liver tissue close to the resection margin with direct injury by cauterization, which led to the highest KC activation levels. The comparison between ROI levels and donor anamnesis indicates that donor conditions and liver tissue quality influence the initial KC activation, although no definitive statement can be made due to the low numbers of donors in each group.

### 3.2. Experimental Setup and Evaluation of Optimal Culture Conditions

In general, cultured KC showed a loss in cell viability associated with a decrease of ROI over 5 d regardless of the type of serum used. The cultivation with FCS had a slightly positive effect on cell viability, while a slight increase of KC activation was observed compared to serum-free cultivation or cultivation with autologous serum (see Supplementary Figure 1 available online at http://dx.doi.org/10.1155/2015/640631).

Cultivation of KC in the presence of the antioxidants *n*-acetyl cysteine or ascorbic acid also showed an increase in the ROI level. However, a beneficial effect on cell viability in comparison to the control was observed during the first 12 h after seeding. N-acetyl cysteine had a stronger effect than ascorbic acid (Supplementary Figure 2).

### 3.3. Compound-Dependent KC Activation

KC can be activated by a variety of stimuli. For determination of the sensitivity of KC towards cell stress, KC were exposed to hepatotoxic drugs (APAP, DIC) or to the known KC activating agent LPS [18]. Of special interest in this experiment was the question, if an additional activation on top of the initial activation is detectable. Therefore, KC cultures were stimulated with varying concentrations of APAP, DIC, and LPS for 24 h. At the end of the activation period, the intracellular ROI levels were measured and normalized to the cell viability.

**Figure 3:**
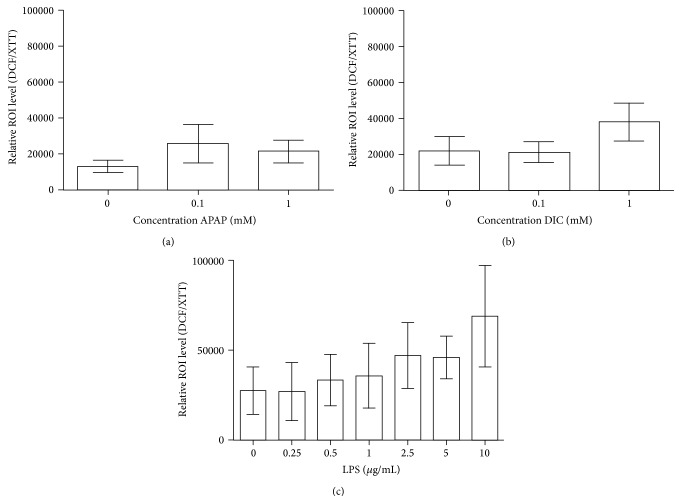
*APAP-, DIC- or LPS-induced stimulation of KC*. APAP (a), DIC (b), or LPS (c) in different concentrations were used for the stimulation of KC for 24 h. The intracellular ROI formation was investigated by the DCF assay. The detected ROI levels were normalized to the cell viability measured by means of the XTT assay. Data show means ± SEM. *N*_LPS_ = 3, *N*_APAP/DIC_ = 5, *n*_LPS_ = 6, and *n*_APAP/DIC_ = 2.

Treatment of KC with APAP for 24 h showed for both concentrations (0.1 and 1 mM) a slight increase in the relative oxidative stress levels by trend (Figure 3(a)). The treatment with DIC for 24 h showed no effect for 0.1 mM DIC and a slight increase in the relative oxidative stress level for 1 mM DIC (Figure 3(b)).

The stimulation with LPS showed a tendency towards a concentration-dependent increase in the relative ROI levels compared to the untreated control (Figure 3(c)). The results showed large variations due to different initial ROI values in activation of KC from individual donors. Taken together, for various concentrations of APAP and DIC, no statistically significant activation was detectable. Furthermore, the positive control LPS did not show any statistically significant KC activation. However, a clear trend for a concentration-dependent activation was observable, which suggests that an additional activation of preactivated KC is possible.

### 3.4. Response of Kupffer Cells to Hepatocyte Stress/Damage

#### 3.4.1. Hepatocyte Stress/Damage after Stimulation with Hepatotoxic Drugs

For the simulation of hepatocyte stress/damage, we treated isolated hepatocytes from 3 different donors (Donor A, Donor B, and Donor C) with the hepatotoxic drugs APAP or DIC (100 *μ*M) for 1 h, respectively. Donor characteristics are detailed in Table 1.

At the end of drug incubation, the DCF assay was performed for the evaluation of potential oxidative stress induction and the XTT assay was performed for assessment of the cell viability. Untreated PHH from the same donor served as control, since they reflect the basal oxidative stress level of the hepatocytes. Due to large variances between the reactions of PHH cultures from individual donors towards the drugs, the results were not merged and are presented on a case by case basis instead.

**Figure 4:**
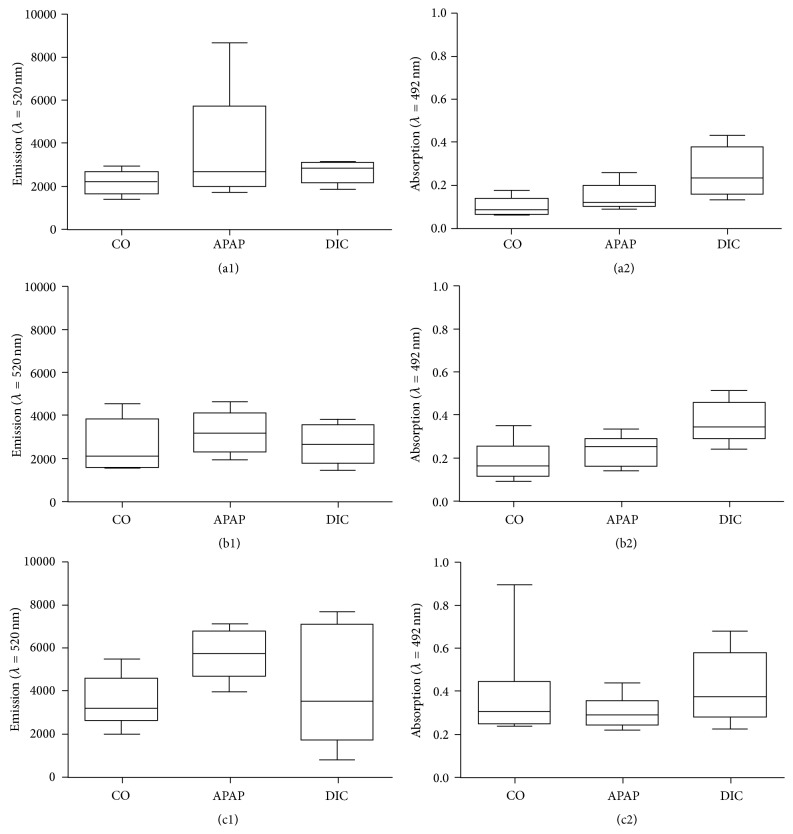
*Induction of oxidative stress in PHH*. PHH from three individual donors (A, B, and C, see Table 1) were treated with 100 *μ*M acetaminophen (APAP) or 100 *μ*M diclofenac (DIC) for 1 h. Intracellular ROI formation ((a1), (b1), and (c1)) was investigated by the DCF assay and the cell activity ((a2), (b2), and (c2)) was determined by the XTT assay. Data are shown as box plots, representing the median, the interquartile range, and minimum and maximum values, *n* = 4.

The investigation of drug-mediated oxidative stress induction in PHH revealed that the basal oxidative stress level varied donor dependently. While donor A and donor B showed similar ROS concentrations in control cultures (Figures 4(a1) and 4(b1)), donor C showed a 1.5 times higher oxidative stress level (Figure 4(c1)). However, all donors showed a low (donor A and B) to moderate (donor C) increase of oxidative stress for APAP, but not for DIC incubation. Investigation of cell viability by means of the XTT assay revealed that stimulation with APAP did not influence the cell viability, while application of DIC resulted in a clear increase in cell activity in donor A (Figure 4(a2)) and donor B (Figure 4(b2)). Donor C (Figure 4(c2)) showed a constant level in cell activity after drug treatment. All three donors showed no decrease in cell activity and therefore no loss in cell viability could be detected.

#### 3.4.2. Kupffer Cell Response to Hepatocyte Stress/Damage

**Figure 5:**
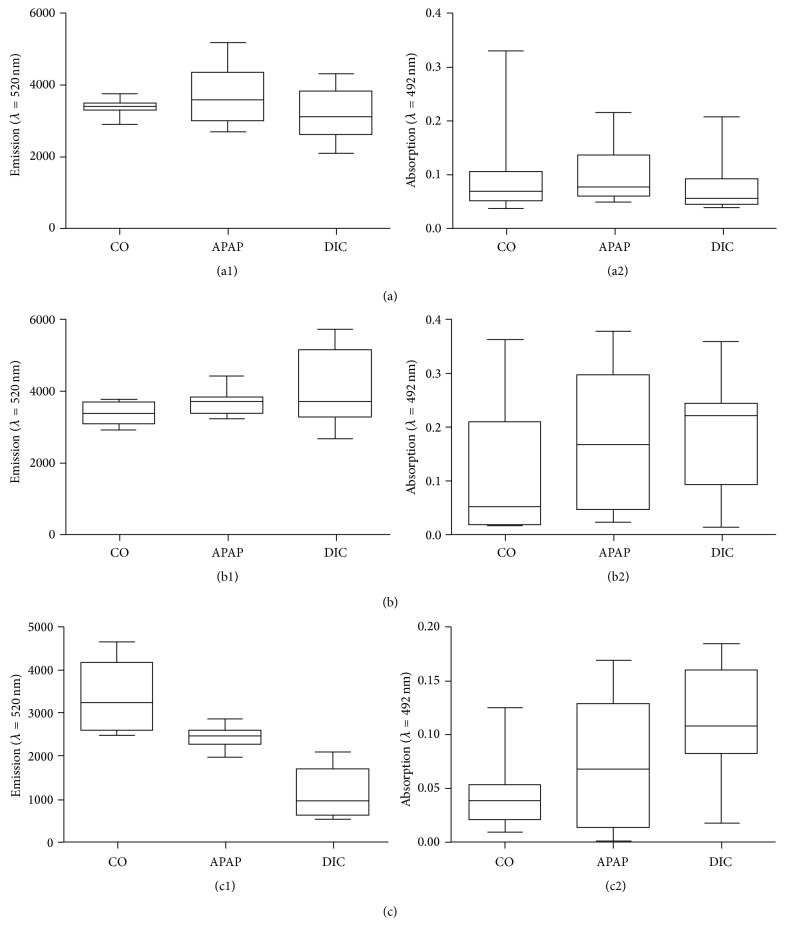
*KC activation with supernatants of PHH previously incubated with subtoxic concentrations of hepatotoxic drugs*. PHH of three donors (A, B, and C, see Table 1) were treated with 100 *μ*M acetaminophen (APAP) or 100 *μ*M diclofenac (DIC) for 1 h. The supernatants were used to stimulate KC from the same donor for 2 h. The intracellular ROI formation ((a1), (b1), and (c1)) was investigated by the DCF assay and the KC activity ((a2), (b2), and (c2)) was determined by the XTT assay. Data are shown as box plots, representing the median, the interquartile range, and minimum and maximum values, *n* = 4.

**Figure 6:**
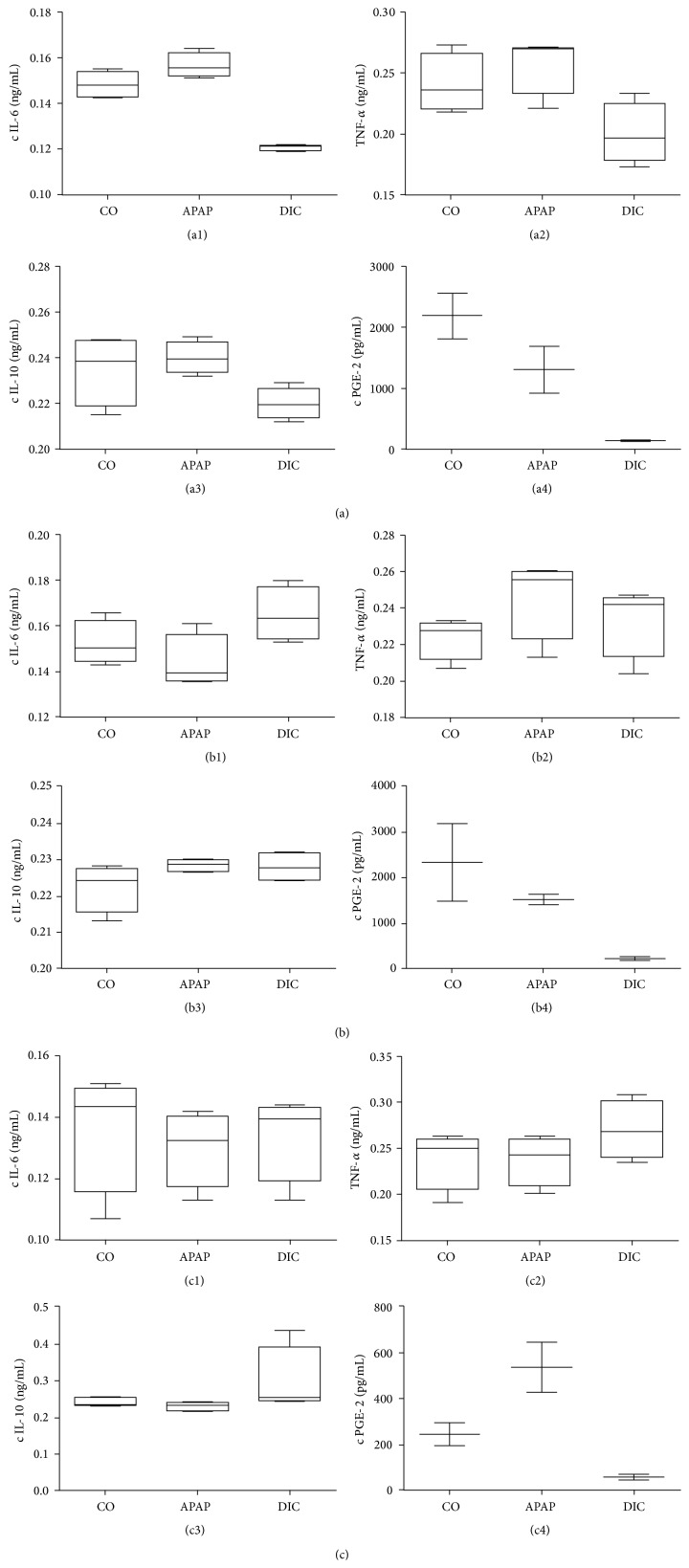
*Secreted cytokine profiles of KC activated with supernatants of PHH previously incubated with subtoxic concentrations of hepatotoxic drugs*. PHH from three donors (A, B, and C; see Table 1) were treated with 100 *μ*M acetaminophen (APAP) or 100 *μ*M diclofenac (DIC) for 1 h. The supernatants were used to stimulate KC from the same donor for 2 h. Inflammatory reactions of KC due to hepatocyte stress/damage were determined by cytokine analysis in KC supernatants. IL-6 ((a1), (b1), and (c1)), TNF-alpha ((a2), (b2), and (c2)), IL-10 ((a3), (b3), and (c3)), and PGE-2 ((a4), (b4), and (c4)) were investigated using cytokine ELISAs. Data are shown as box plots, representing the median, the interquartile range, and minimum and maximum values, *n*_(IL-6,TNF-alpha,IL-10)_ = 4  *n*_(PGE-2)_ = 2.

To evaluate the immunological response of KC after hepatocyte stress/damage, the isolated KC were stimulated with the supernatants of PHH having been pretreated with hepatotoxic drugs (see Section 3.4.1). To evaluate the KC activation, the cell activity and intracellular ROI formation, the KC response and the cytokine secretion were measured, respectively. The cell activity increased compound dependently in KC from all three tested donors (Donor A, Donor B, and Donor C) after stimulation with supernatants from drug-treated PHH. The corresponding ROI formation and the cytokine release as markers for KC activation and response, respectively, showed donor- and compound-dependent signals (Figures 5 and 6). Due to these very individual reactions, the donors were investigated on a case by case basis.

Donor A was a young healthy woman with a benign liver tumor (Table 1). The activation of KC increased slightly after stimulation with supernatants from APAP-treated PHH, compared to the untreated control. In contrast, there was no increase in cell activity. KC stimulation with DIC did not lead to any changes neither in the activation measured by intracellular ROI formation nor in cell activity (Figure 5(a)).

Cytokine release of KC after stimulation with APAP-treated PHH showed a slight increase in cytokines IL-6 and TNF-*α* but no change in the IL-10 level compared to the KC stimulation with control hepatocytes. Supernatants from DIC-treated PHH evoked a notable decrease of the secretion of IL-6 and TNF-*α* in KC cultures. The PGE-2 release in donor A was comparable to the release in donor B. In both donors, PGE-2 release decreased drug dependently, whereas the effect of KC stimulated with DIC-treated PHH was higher than after APAP treatment (Figures 6(a) and 6(b)).

Donor B was a 50-year-old man who suffered from a colorectal liver metastasis (Table 1). The stimulation of KC with the supernatants of DIC- or APAP-exposed PHH led to an elevated KC activation quantified by an increase in the ROI level and in cell activity (Figure 5(b)).

Regarding the KC reaction, the stimulation with APAP-treated PHH showed a slight increase in the release of TNF-*α* comparable to donor A but no effect on the level of IL-6. After KC stimulation with supernatant from DIC-treated PHH beside an increase of TNF-*α*, an increase of the IL-6 concentration was measured (Figure 6(b)). In terms of IL-10 secretion, no differences were detected between KC incubated with supernatant from compound-treated PHH and those from control hepatocytes (Figure 6(b)).

Donor C was a 57-year-old man with diabetes suffering from a Klatskin tumor. This rare form of cholangiocellular carcinoma is closely connected to cholestasis and therefore to a stress/damage to the affected liver tissue. Additionally, the patient had previously undergone portal vein embolization (Table 1). The results of this donor were different from those of the other two investigated donors. The KC activity measured by changes in intracellular ROI levels was clearly decreased after stimulation with supernatant from drug-treated PHH compared to the stimulation with that of control hepatocytes. This decrease in KC activation was more intense after the stimulation with DIC-treated PHH compared to the stimulation with APAP-treated PHH (Figure 5(c)). The effect on the KC activity was inverse. Here, a strong increase in cellular activity was detected after stimulation of KC with both compounds.

The KC reaction after stimulation with supernatant from APAP-treated PHH showed no changes in TNF-*α*, IL-6, and IL-10 secretion compared to stimulation with untreated PHH. In contrast, the PGE-2 secretion increased noticeably after treatment with APAP-stimulated PHH. DIC-treated PHH induced an increase in the release of IL-10 and TNF-*α* in KC. After stimulation of the KC with supernatant from DIC-treated PHH, the PGE-2 levels decreased comparably in comparison to the other investigated donors. The IL-6 level remained unchanged (Figure 6(c)).

## 4. Discussion

DILI is responsible for severe patient morbidity and mortality [31] and, in consequence, causes massive economic losses in pharmaceutical industry [2]. While different mechanisms of action are described, there is evidence that the involvement of immunologic reactions leading to sensitization reactions and autoimmune diseases might play a major role [32]. Previous* in vivo* preclinical testing strategies failed due to the idiosyncratic nature of DILI causing its low frequencies. The idiosyncrasy is a result of immunologic reactions in hepatic inflammation leading in most cases to immune tolerance towards drug-mediated hepatotoxicity. To bypass systemic tolerance reactions, we hypothesize that a DILI risk could be detectable in an* in vitro* liver model, which enables the investigation of immunologic cell-cell communication at an early stage of the hepatotoxic event. Aim of the present study was the establishment of a human* in vitro* model for the simulation of hepatic tissue stress/damage, which allows for the investigation of immune-mediated signaling in hepatic inflammatory reactions.

KC are the first cells confronted with a hepatic tissue damage. These tissue-resident macrophages sense tissue damage and cell stress, process incoming signals, and communicate a reaction to other NPC and to the systemic immune system [20, 24, 33].

Therefore, we established a liver model consisting of PHH being responsible for displaying the hepatotoxic event and KC for mediating immunologic cell-cell communication. In the present study, PHH and KC were successfully isolated from resected human liver tissue samples. An optimized separation procedure using an adherence separation step allowed for the isolation of KC in a high quantity and purity. KC were clearly identified by detection of the macrophage-specific surface protein CD68 and by their ability for phagocytosis.

The determination of intracellular ROI formation revealed that KC are already partially activated after isolation (initial KC activation). Activated KC produce ROI as part of the NF-*κ*B signaling pathway, which can be used as a marker for the determination of the KC activation [34]. Lowest KC activation levels were measured in healthy liver tissue from young donors with benign tumors and no secondary diseases or interventions. KC can be activated by various endogenous sources arising from tissue damage, like intracellular components, cell debris released by necrosis, apoptotic bodies [35], and inflammatory cytokines [36]. This was reflected in KC isolated from liver tissue containing a resection border with freshly damaged and destroyed liver tissue. Diseases and medical interventions leading to hepatic tissue damage may consequently increase the initial KC activation. We suggest that the correlation of donor anamnesis to the KC activation level is proportional to the time span since induction and intensity of the tissue damage, respectively. The testing of different media supplements to reduce the initial activation showed that KC activation is not reversible under the conditions used, although the use of FCS or of FCS in combination with *n*-acetyl cysteine showed both beneficial effects on KC viability.

However, the results from the additional KC activation by LPS confirmed that KC can be used for the detection of inflammatory events when compared to untreated control cultures. Therefore, an additional activation of KC is possible and can be quantified by using ROI as a marker for Kupffer cell activation. Using ROI as a marker for KC activation was demonstrated previously by Uchikura et al. and by Hosomura et al. [18, 30]. In consequence, KC can be used for the experiments on drug-mediated hepatocyte damage and its effect on KC.

The evaluation of the response of KC to drug-mediated stress/damage in hepatocytes was tested in cultures from three different donors in two steps: in the first step, PHH were incubated with hepatotoxic compounds (APAP or DIC). The investigation of drug-mediated oxidative stress revealed APAP but not DIC-induced oxidative stress in all three donors. These results are in accordance with those from other studies showing higher ROS induction for APAP compared to DIC [26, 37]. However, the evaluation of cell viability evaluated by measurement of the cell activity showed no decrease in energy metabolism in PHH cultures after compound treatment. Therefore, the short incubation time and low concentration represent subtoxic conditions, which can induce cell stress, but did not lead to irreversible toxic effects. Moreover, we observed that the amplitude of cell stress was donor-dependent with age and preexistence of a liver damage.

In contrast, incubations of APAP and DIC in KC cultures from three different donors for 24 h showed no statistically significant KC activation. These results were plausible due to the requirement of CYP450 isoenzymes for the hepatotoxic mode of action of APAP and DIC, which were less expressed in KC [38]. However, a KC activation for 100 *μ*M of APAP and for both compounds for 1 mM concentration was observable, though not statistically significant. Accordingly, we used 100 *μ*M in our main study. The drug is first metabolized by the PHH before coming in contact with KC. Therefore, far lower concentrations of remaining parent compounds are expected in the transferred supernatants. Additionally, we used much lower incubation times. Therefore, a direct effect of hepatotoxic compounds on KC from remaining compound in the supernatants of drug-treated hepatocytes is unlikely.

In the second step, transfer of the supernatant of drug-treated PHH cultures to corresponding KC cultures revealed a donor- and compound-dependent activation of KC (see overview in Table 2). In general, we observed an increased KC activity correlating with the vulnerability of donors to hepatotoxicity as seen by APAP-mediated ROS induction. The KC activation measured on the signaling level demonstrated a change in ROI formation interpreted as pro- or anti-inflammatory signaling.

The readout for ROS measurement in PHH and KC following activation was partly very low and showed an obvious change only for one donor. Choosing subtoxic conditions (100 *μ*M) to induce cell stress rather than cell death required short incubation times and low drug concentrations [26]. Hepatotoxic effects in this range of drug concentration are rather mild as shown in 3D coculture liver models [11]. However, the observed donor- and compound-dependent activation was confirmed by the measurement of specific pro- and anti-inflammatory cytokines (see overview in Table 2). We observed a proinflammatory KC reaction when supernatant from stressed/damaged hepatocytes from healthy donor tissue was used. In contrast, an increased concentration of anti-inflammatory cytokines was detected if the supernatant was obtained from liver tissue with a preexisting liver damage. These results indicate that PHH from older and diseased donors are more vulnerable to toxic compounds than PHH from younger donors. This is in accordance with observations demonstrating that detoxification capabilities decrease with ongoing age [39]. While healthy donors showed tendencies towards proinflammatory reactions a clear anti-inflammatory reaction was observed in a donor with preliminary tissue damage due to cholestasis as a result from his tumor. This finding is in accordance with studies showing that, in chronic or preexisting liver damage, KC silence an additional inflammatory signal to avoid overreaction [33, 40, 41]. The observed compound-specific effects could be due to hapten formation in case of DIC-mediated hepatotoxicity, which has a stronger impact on KC activation and reaction than cell stress mediated by ROS formation from APAP. In this context, KC are capable of detecting drug-mediated cell stress at an early stage even before cell damage by hepatotoxicity occurs. In our experimental setup, only soluble mediators from the PHH supernatant can be responsible for the induction of the immunologic reactions in KC, like, for example, cytokines, endogenous proteins, and haptens. Experiments using hepatotoxic compounds and their corresponding drug-protein adducts have revealed a regulating role of KC [33]. Other studies showed KC activation by cytokines released as a response to different compounds, like, for example, oncostatin [24], LPS [18, 20], or HCV-related proteins [30]. These data confirm that KC allow for detection of immunological signals as a first reaction to drug-mediated hepatocyte stress/damage.

The low number of cases of this study is a major limitation even though the results were evaluated by means of different correlating readout parameters. Additionally the use of primary human cells led to large variations in some experiments, for example, the LPS stimulations. Even if we observed clear trends in our results, statistical significance is missing in some cases. We conclude that KC related immunologic reactions are donor-specific and that the complex* in vitro* model consisting of primary human cells is influenced by many patient-related factors. Therefore, the results have to be considered on a case by case basis until further donors are investigated to validate these data.

DILI is described as an idiosyncratic reaction towards specific drugs and its prediction is difficult [6]. The results from this study suggest that using KC as detector cells a hepatotoxic risk can be estimated and reflects compound- and donor-specific effects. Moreover, this hepatotoxic stress is also measurable when subtoxic concentrations of hepatotoxic drugs are investigated. This is the first study using human KC for detection of hepatotoxic stress/damage induced by DILI compounds. It is known that existing* in vitro* models for the investigation of hepatotoxicity using PHH monocultures are not capable of reflecting the* in vivo* toxicity. The models suffer in general from the need of much higher concentrations of toxic drugs to induce hepatotoxicity compared to the* in vivo* situation. The effect on ROS induction leading to cell stress and KC activation observed for APAP was less pronounced in comparison to DIC. We suggest that DIC tends to formation of protein adducts rather than ROS induction. Classical hepatotoxicity testing measuring cell viability and oxidative stress does not capture a DILI risk in this case. Therefore, cocultures of PHH and KC could be used as a tool for evaluation of a DILI risk based on different mechanisms of actions. Using liver cells from different donor groups would also allow for the investigation of donor-specific effects. Thus, our established liver model is a useful tool for the investigation of hepatotoxic effects of DILI compounds and could contribute to an improved drug safety in drug development.

## Supplementary Material

For optimization of KC culture conditions, different serums and antioxidant supplementation were tested. In general, cultured KC showed a loss in cell viability associated with a decrease of ROI over 5 d regardless of the type of serum used. The cultivation with FCS had a slightly positive effect on cell viability, while a slight increase of KC activation was observed compared to serum-free cultivation or cultivation with autologous serum (Supplementary Figure 1). Cultivation of KC in the presence of the antioxidants acetyl cysteine or ascorbic acid also showed an increase in the ROI level. However, a beneficial effect on cell viability in comparison to the control was observed during the first 12 h after seeding. N-acetyl cysteine had a stronger effect than ascorbic acid (Supplementary Figure 2).

## Figures and Tables

**Table 1:** Anamnesis data of investigated donors.

	Diagnosis	Sex	Age	BMI	Notes
Healthy	Adenoma	F	48	29	—
	Cholangiocellular carcinoma	F	50	20	—

Multimorbidity	Klatskin tumor	M	80	31	Hypercholesterolemia
	Colorectal liver metastasis	M	72	23	Coronary heart disease
	Cholangiocellular carcinoma	M	74	29	Diabetes, hypertension, and in situ split
	Gall bladder carcinoma	F	57	28	Portal vein embolisation, hypertension, and cholestasis
	Cholangiocellular carcinoma	F	75	24	Hypercholesterolemia
	Cholangiocellular carcinoma	M	61	31	Hypertension, hypercholesterolemia
	Hemangioma	F	43	28.4	Diabetes
	Cholangiocellular carcinoma	M	47	23	Terminal renal failure, hypercholesterolemia

Chronic inflammation	Klatskin tumor	M	72	24	Chronic inflammation, diabetes, and hypertension

Portal vein embolisation	Colorectal liver metastasis	F	60	—	Portal vein embolisation
	Colorectal liver metastasis	M	57	28	Portal vein embolisation, chemotherapy

Cholestasis	Cholangiocellular carcinoma	F	72	22	Cholestasis
	Cholangiocellular carcinoma	F	77	22	Cholestasis

Chemotherapy	Colorectal liver metastasis	F	71	—	Chemotherapy

Hepatic steatosis	Hepatocellular carcinoma	M	75	—	Resection area, steatohepatitis
	Adenoma	F	32	—	Diabetes, steatohepatitis

Resection area	Hemangioma	M	47	27	Resection area
	Cholangiocellular carcinoma	M	62	25	Resection area, chemotherapy

Donor A	Focal nodular hyperplasia	F	19	22	—

Donor B	Colorectal liver metastasis	M	50	23	Diabetes, smoker

Donor C	Klatskin tumor	M	52	25	Diabetes, portal vein embolisation

**Table 2:** Summary of KC activation and reaction.

Donor	Drug tested	ROI formation	Cell activity	IL-6	TNF-a	IL-10	PGF-2
A	APAP	↑	—	↑	—	—	↓
	DIC	—	—	↓↓	↓	—	↓↓

B	APAP	↑	↑	—	↑	—	↓
	DIC	↑↑	↑	↑	↑	—	↓↓

C	APAP	↓	↑	—	—	—	↑
	DIC	↓↓	↑↑	—	↑	↑	↓↓

↑: slight increase, ↑↑: increase, ↓: slight decrease, ↓↓: decrease, and —: no change.

All data were compared to the control.
