# Supplementary material for: Subtoxic Concentrations of Hepatotoxic Drugs Lead to Kupffer Cell Activation in a Human In Vitro Liver Model: An Approach to Study DILI
Source: Mediators Inflamm. 2015 Sep 28;2015:640631. doi: 10.1155/2015/640631 (PMC4600928; doi:10.1155/2015/640631)
Supplement: Supplementary file 1 — For optimization of KC culture conditions, different serums and antioxidant supplementation were tested. In general, cultured KC showed a loss in cell viability associated with a decrease of ROI over 5 d regardless of the type of serum used. The cultivation with FCS had a slightly positive effect on cell viability, while a slight increase of KC activation was observed compared to serum-free cultivation or cultivation with autologous serum (Supplementary Figure 1). Cultivation of KC in the presence of the antioxidants acetyl cysteine or ascorbic acid also showed an increase in the ROI level. However, a beneficial effect on cell viability in comparison to the control was observed during the first 12 h after seeding. N-acetyl cysteine had a stronger effect than ascorbic acid (Supplementary Figure 2). [file 640631.f1.pdf]

Supplements

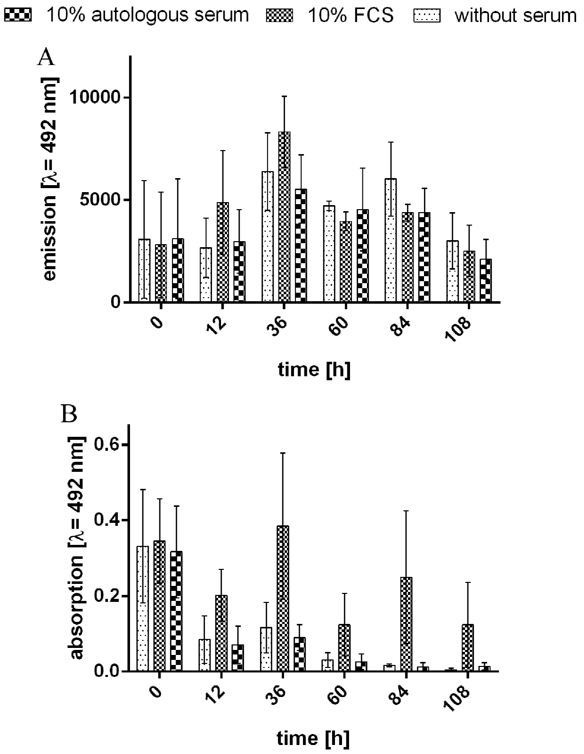

Supp.-Fig. 1 Testing of different cell culture supplements for KC culture. KC were cultured for up to 108 h using RPMI medium supplemented with 10% autologous serum, 10% FCS or without serum. The intracellular ROI formation (A) was investigated by the DCF-assay and the mitochondrial activity (B) by the XTT-assay. Data show means ± SEM. N=3; n=3

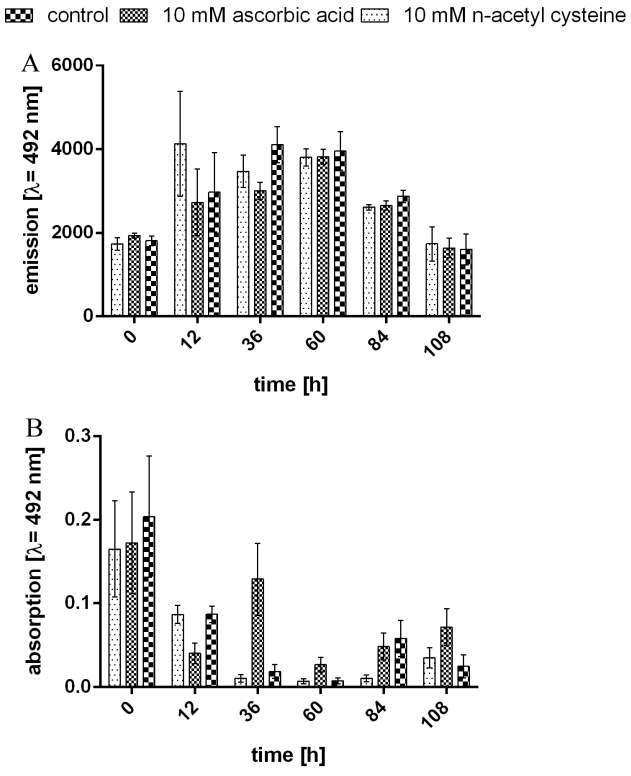

Supp.-Fig. 2 Testing of antioxidant supplementation to reduce initial KC activation. KC were cultured for up to 108 h using RPMI medium supplemented with or without 10 mM n-acetylcysteine or 10 mM ascorbic acid. The intracellular ROI formation (A) was investigated by the DCF-assay and the mitochondrial activity (B) by the XTT-assay. Data show means ± SEM. N=3; n=3
